# Antioxidant and Anticancer Roles of a Novel Strain of* Bacillus anthracis* Isolated from Vermicompost Prepared from Paper Mill Sludge

**DOI:** 10.1155/2018/1073687

**Published:** 2018-08-26

**Authors:** Ram Kumar Ganguly, Sujoy Midya, Susanta Kumar Chakraborty

**Affiliations:** Department of Zoology, Vidyasagar University, Midnapore 721102, West Bengal, India

## Abstract

Mass production of vermicompost using suitable species of earthworms and selecting target organic waste materials has appeared to be a great development in the realm of biotechnological research for the sustainable eco-management. Although, for the bioconversion of organic wastes to vermicompost, suitable earthworm species play major roles, a hoard of bacterial assemblages by virtue of production of different enzymes facilitate the process of vermicomposting. The present study has documented the roles of vermicompost associated bacteria in combating, preventing, and controlling of cancer so as to open a new vista not only in the field of vermitechnology but also on biomedical research. Earthworms' associated bacterial metabolic products having their unique physicochemical excellence have gained importance due to their roles as a facilitator of apoptosis (programed cell death in a MCF-7 cell line). The antioxidant and anticancer activities of ethyl acetate extracts' of vermicompost associated bacterium* Bacillus anthracis* were undertaken by antioxidant assay which revealed maximum DPPH radical scavenging effect (75.79 ± 5.41%) of the extracts' at 9 00 *μ*g ml-1. Furthermore, the crude extracts obtained from the same bacteria were found to decrease the activity of SOD (superoxide dismutase) with the increase in doses. MTT assay showed potent cytotoxic activity against human breast adenocarcinoma cells (MCF-7) with the IC50 value of 46.64 ± 0.79 *μ*g ml-1. It was further confirmed through Hoechst 33258 staining of nuclear fragmentation assay and DNA fragmentation analysis. Western blotting test has confirmed a downregulation of Akt upon application of crude extracts. Increase of SOD activity along with decrease of Akt level reflects that the mode of action is entirely PI-3K dependent. This study tends to indicate that* B*.* anthracis* isolated from vermicompost could be potentially explored for the development of new therapeutic agents, especially against cancer.

## 1. Introduction

Vermitechnology involving vermiculture and vermicomposting process has emerged as a highly suitable, user-friendly, and cost-effective eco-technology for proper organic waste management [1]. During vermicomposting, nutrients such as nitrogen, carbon, potassium, phosphorus, zinc, and calcium of waste materials while passing through the earthworms gut are homogenized in bacterial rich environment into a highly mineralized chemical forms which are much more available to the plants when used as fertiliser [2]. It is a decomposition process in which biochemical degradation of the of organic waste materials as substrate occurs by the joint action of earthworms and microorganisms by way of fragmentation, conditioning, and stabilization [3]. Although the process involves microbial degradation, earthworms are the actual drivers of this technology [4, 5].

The present study has attempted to highlight a new vista on roles of these microbes in the context of health science. Microorganisms have so far established their importance in combating several medical problems through the production of several bioactive compounds for therapeutic purposes [6, 7]. Each year millions of people are diagnosed worldwide with cancer, and more than half of these patients eventually die from this disease. Based on global cancer statistics published in the year 2011, 12.7 million cases of cancer were detected and 7.6 million cancer deaths in a year were reported [8, 9]. Conventional cancer treatments such as surgery, chemotherapy, and radiotherapy often fail to achieve a complete cancer remission. Moreover, it has been widely recognized that radiotherapy or chemotherapy is likely to cause significant side effects [10]. This fact has prompted to undertake more researches for the development of patient-friendly new approaches for the treatment of cancer. Microbial based therapy of cancer is one of the emerging cancer treatment modalities [11]. Important advances have been made to study and develop live bacteria or bacterial products such as proteins, antioxidant enzymes, immune toxins, and secondary metabolites which specifically target cancer cells and cause tumor regression through growth inhibition and arresting of cell proliferation in order to arrest and retard apoptosis induction [12, 13]. Recently, many studies have focussed on and recommended the anticancer property of bacterial metabolites of* Clostridium*,* Bifidobacterium*,* Listeria*,* Salmonella*,* Lactobacillus*,* Escherichia,* etc. [14, 15].

In such context, the present study has opened up a new vista towards the utility of vermicompost as a potential incubator for some beneficial bacteria which not only trigger and accelerate the process of vermicomposting but also paved the way of utilizing them as anticancer agents.

## 2. Materials and Methods

### 2.1. Collection of Sludge

Sludge sample was collected in plastic bags from UNIGLOBAL paper mill at Jhargram, India (22° 27′ 0′′ N, 86° 59′ 0′′ E) under aseptic conditions. Samples after being processed and sundried were used for vermicomposting.

### 2.2. Experimental Design and Chemical Analysis

Sundried samples after being properly grounded were mixed up with supplements such as cow dung and straws in a ratio of 5 : 4 : 1 (ratio was optimized focussing on least mortality rate of earthworms) and were consumed and turned over by* Eisenia fetida *for a duration of 60 days. Sludge samples could not be used directly due to its toxic properties. The temperature during the entire process of composting was maintained at 25°C with a moisture content of around 60% and pH (7.4-7.8) through the regular sprinkling of water. All the experiments were performed in triplicate [16].

### 2.3. Growth of Bacteria and Preparation of Crude Extract

1.0 gm of the sample from vermicompost of each replicate was subjected to serial dilution up to 10^−6^ in normal saline solution. 200 *μ*l of the sample was added in nutrient broth (HIMEDIA) and kept at incubation for 48 h at 37°C. 10 *μ*l of samples was taken and further spread over minimal agar plate made up of nutrient agar (HIMEDIA) and kept at incubation for 24 hrs at 37°C for proper isolation of colonies [16].

## 3. Identification of Bacteria

### 3.1. 16srRNA Gene Sequencing and Phylogeny Construction

16srRNA sequencing technique was used here for identification of bacterial isolates. Three heat shock cycles (20 min at -85°C and 15 min at 95°C) were used for genomic DNA isolation. Amplification of DNA samples through PCR (polymerase chain reaction) was carried out using two universal primers 27F (5′-AGAGTTTGATCMTGGCTCAG-3′) and 1492R (5′-AGAGCCCGATCMTGGCTCAG-3′). Sequencing of DNA sample was performed using ABI PRISM Big Dye TM Terminator Cycle Sequencing Kits with AmpliTaq DNA polymerase (FS enzyme) (Applied Biosystems). Single-pass sequencing was performed using 27F/1492R primers and fluorescently label fragments were purified and subjected to electrophoresis in an ABI 370X1 sequencer (Applied Biosystems) [16, 17].

16srRNA gene sequence was BLAST using NCBI BLAST search tool. MUSCLE 3.7 programs used for multiple alignments of sequence and were cured using program G blocks 0. 91b. Finally, PhyML3.0Alrt was used for phylogeny analysis using HKY85 as substitution model and program Tree Dyn 198.3 was used for tree rendering [18].

### 3.2. Preparation of Crude Extract

Bacterial isolate was cultured in nutrient broth at 35±2°C for 72 h and then it was centrifuged at 10000 rpm for 20 min. Supernatant was collected and filtered using 0.22 *μ*m filter to remove bacterial cells. Cell free supernatant was allowed to mix with ethyl acetate in ratio of 1 : 1 and was concentrated using rotary vacuum evaporator. Crude extracts from the produced vermicomposts were obtained and used for further experiments [19].

### 3.3. Antioxidant Activity

#### 3.3.1. DPPH Radical Scavenging Activity

The capability of crude extract to demonstrate antioxidant activity was determined through DPPH (1,1-diphenyl-2-picrylhydrazyl) radical scavenging assay [20]. Different concentrations (100, 300, 600, and 900 *μ*g mL^−1^) of extracts were prepared in distilled water. Ascorbic acid (100, 300, 600, and 900 *μ*g mL^−1^) was used here as positive control. 0.15 mM of DPPH solution was added to both test sample and control. It was then vortexed for 5 minutes and allowed to stand for 20 min at room temperature in dark. Absorbances of the samples were measured in spectrophotometer (SHIMADZU; UV–1601) at 517 nm. Radical scavenging activity was determined using the following equation: Scavenging effect (%) = [1- (*A* sample–*A* sample blank)/*A* control] x 100

*A* control is the absorbance of the control,* A* sample is the absorbance of the extract, and* A* sample blank is the absorbance of the sample only.

#### 3.3.2. Activity Staining of SOD

Activity staining of SOD (Superoxide Dismutase) was performed using nondenaturing PAGE, followed by staining of gel. Protein from each sample was separated by 10% nondenaturing Polyacrylamide Gel Electrophoresis at 4°C. After electrophoresis, the gel was soaked in 1.23 mM NBT (Nitro-blue Tetrazolium) solution for 20 min in dark. The gel was briefly washed in distilled water and incubated for 15-20 min under dark in 100 mM phosphate buffer (pH 7.0) containing 28 mM TEMED (Tetramethylethylenediamine) and 0.28 mM riboflavin. The gel was exposed to a fluorescent light until the appearance of clear zones of activity bands with blue background. Intensity of bands was analyzed by densitometry scanning using an Alpha Image Analyzer System (Alpha Innotech, San Leandro, CA, USA) [21].

### 3.4. Anticancer Activity

#### 3.4.1. MTT Cytotoxicity Assay

The assay was used to determine the anticancer activity of crude extract over MCF-7 (human breast adenocarcinoma) cancer cell line. Cells were grown as monolayer in DMEM-AT068 with necessary supplementation at 37°C under 95% humidified air and 5% CO_2_. In brief, cells at concentration of 3 x10^3^ cells per well were seeded in 100 *μ*l of complete medium with various concentrations (50, 100, 150, and 200 *μ*g mL^−1^) of crude extracts. After that 10 *μ*l of 5 mg per ml MTT [3-(4,5-dimethylthiazol-2-yl)-2,5-diphenyltetrazolium bromide] was added in each well for 3 h and was kept at dark. 100 *μ*l of DMSO was added after removing of media and absorption of formazan solution was measured at 570 nm using a microplate reader (ECIL). Cytotoxicity of the crude extract was measured as IC50 value (concentration of extract inhibiting 50% of cell growth) [22].

#### 3.4.2. Hoechst 33258 Staining

Single cover slip was placed in each well of 6-well microtitre plate and subsequently monolayer of MCF-7 cell culture was trypsinized. The number of cells was adjusted to 50000 cells/mL and 2.5 mL of cell suspension was added drop by drop on cover slip in each well. The control well contained only maintenance medium. The plates were incubated at 37°C in 5% CO2 atmosphere [23]. After this, incubation media was removed and the cells were washed with PBS for two times. Staining was done Hoechst 33258 (5 *μ*g/ml in PBS) for 25 minutes and after extensive washing by PBS cells were visualized through fluorescence microscope (Motic) at 40X objective magnification.

DNA was extracted from MCF-7 cells. The liver cells were treated with 25 mMTris–HCl buffer (pH 7.5) containing 0.5% SDS, 0.5 mg/mL proteinase K, and 5 mM EDTA at 55°C for 1 h. Methodology was followed according to Riebiero et. al. [24].

### 3.5. Western Blotting

Western blotting is often used to separate and identify proteins [25]. 60 *μ*g of protein sample was denatured in gel loading buffer in boiling water bath for 5 minutes. The samples were cooled on ice and loaded on 10% SDS poly-acrylamide gel along with protein marker. Electrophoresis was carried out at 15 mA in stacking gel and resolved at 30 mA in resolving gel. After electrophoresis, protein was transferred into PVDF membrane overnight at 4°C with constant power supply of 50 V. Protein transfer was confirmed through Ponceau S staining. Then membrane was blocked in 5% nonfat milk in PBS (pH 7.4) for 2 h at room temperature. Blot was then incubated with primary antibody P-AKT ser 473 (1 : 1000 dilution) in 1% BSA and 0.05% Tween 20 in PBS (pH 7.4) overnight at 4°C. After three washes with 1X PBST for 5 min, blot was incubated with HRP conjugated secondary antibody (1 : 2500 dilution) in PBS (pH 7.4) containing 1% BSA and 0.05% Tween 20 for 3-4 hours in rocker at room temperature. After washing with PBST (pH 7.4), immune reactive protein was detected using ECL super signal kit (Pierce Biotechnology Rockford, IL) on X-ray film. Intensity of the band was analyzed by densitometry scanning. Relative densitometry values have been calculated after normalization with *β* actin.

### 3.6. Statistical Analysis

Statistical analysis was performed by SPSS software using one-way analysis of variance followed by Tukey test. Pairwise T-test was made in comparing values of treated cells with respect to normal. Values are being expressed as mean ± SEM obtained from different sets of experiments. Significance of the tests was considered with P value at P = 0.05. All the experiments were performed in triplicate.

## 4. Results

### 4.1. Isolation of Bacteria and Phylogenetic Affiliation

**Figure 1:**
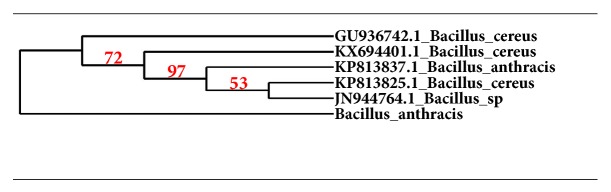
Phylogenetic affiliation of* Bacillus anthracis*.

Different colonies of bacteria were isolated and upon 16srRNA sequencing they were found to be a strain of* Bacillus anthracis.* Previously, this bacterium was isolated from environmental sample like soil [Figure 1] [26].

### 4.2. DPPH Radical Scavenging Activity

**Figure 2:**
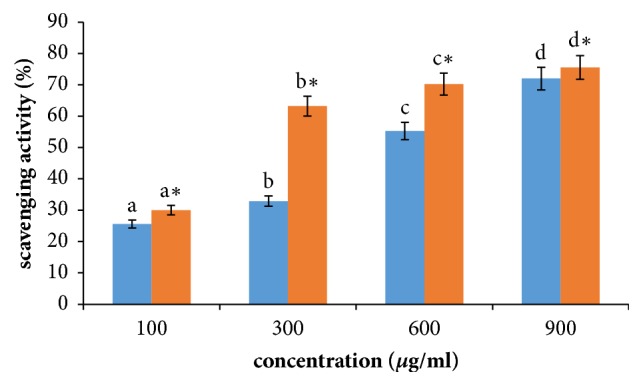
Effect of crude bacterial extracts on scavenging activity in control and treated MCF-7 cells. Results are expressed in terms of values representing mean ± SD where n=3 at ^*∗*^P = 0.05.

Though there are many antioxidants available from plants and other food sources but antioxidants from bacterial sources due to their efficacies ruled out others [27]. DPPH radical is scavenged by antioxidants and is converted to reduced DPPH. Linear progression of activity in both experimental and control set-ups reflect that the extracts obtained from* B. anthracis *can perform as natural antioxidant with high efficiency. These findings have established the facts that extracts are good proton donors and hence can be used as free radical inhibitors. Differential activity of extracts is supposed due to the contribution of different chemical components having varied concentrations of biometabolites. Role of bacterial extracts as antioxidants in terms of DPPH scavenging was established earlier over* B. Subtillis*,* Streptomyces*,* Virgibacillus*, etc. [Figure 2] [27–30].

### 4.3. MTT Assay

**Figure 3:**
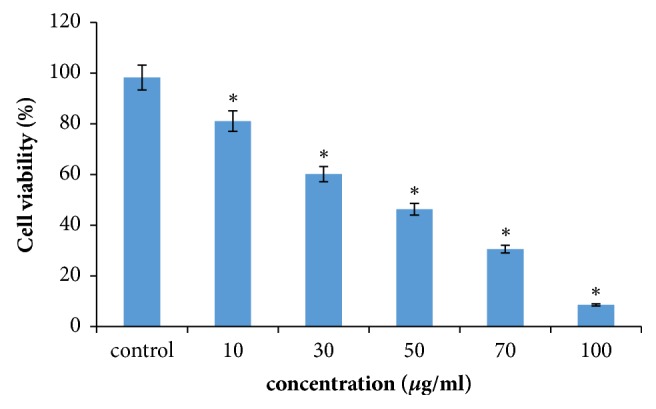
Effect of crude bacterial extracts on cytotoxic activity in control and treated MCF-7 cells. Results are expressed in terms of values representing mean ± SD where n=3 at ^*∗*^P = 0.05.

MTT cytotoxicity tests were conducted to identify the anticancer property or cytotoxicity of extract against MCF-7. It reflects a strong cytotoxicity at IC50 value of 64.65 *μ*g ml after an incubation of 48 h. Metabolites with lower IC50 value prove to be efficient in cytotoxicity even at low dose [31]. Active principles of cellular toxicity resides on the bioactive metabolites of the extracts. The present findings corroborate earlier anticancer study of crude extracts against MCF-7 cells and demonstrate a potent cytotoxicity at lower IC50 value. A good cytotoxic activity against He La cells was found with the application of crude extracts of* B. thuringiensis *isolated from mangrove sediments [32, 33]. A cell line specific activity of cytotoxicity as noticed in the present study using bacterial extracts is supposed to be due to the presence of secondary metabolites as well as complexity in signal cascade guiding for apoptosis [Figure 3.].

### 4.4. Nuclear Fragmentation and DNA Fragmentation Assay

**Figure 4:**
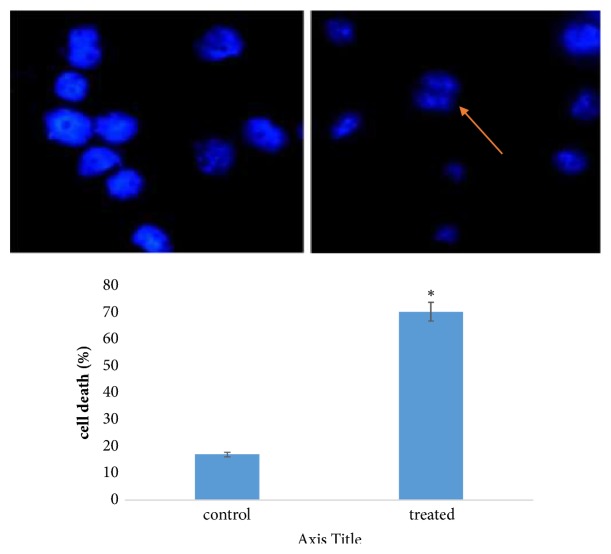
Effect of crude bacterial extracts on nuclear fragmentation in control and treated MCF-7 cells. Results are expressed in terms of values representing mean ± SD where n=3 at ^*∗*^P = 0.05.

Hoechst 33258 is a DNA specific dye which stains chromatin present in nucleus of cell. Nuclear morphology can easily be visualized through this staining method. In this experiment, some hallmarks of apoptosis related to nucleus like nuclear condensation and fragmentation have been observed; nuclear fragmentation analysis further confirms its potential as anticancer compound [Figure 4] [34, 35].

**Figure 5:**
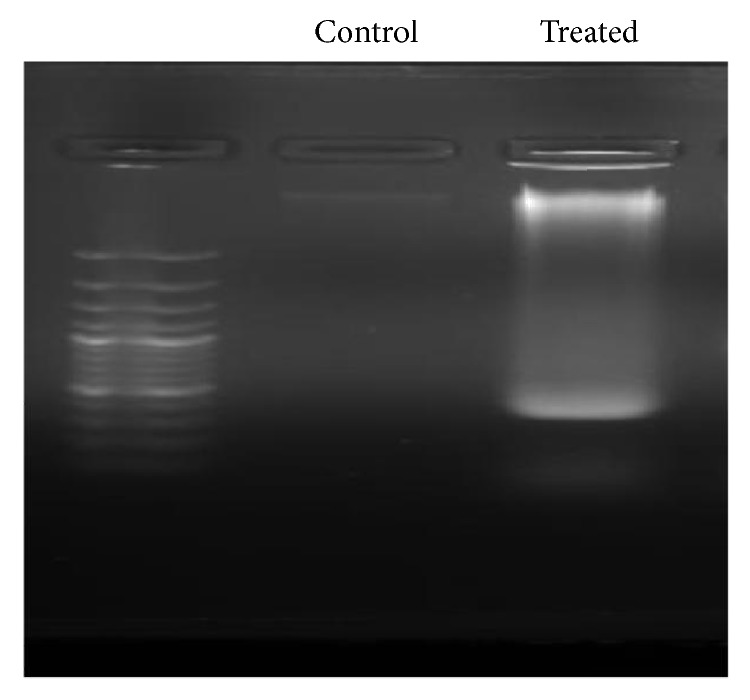
DNA fragmentation assay of ethyl acetate extract treated MCF 7 treated cells with respect to control.

DNA degradation of MCF-7 cells was noticed upon application of ethyl acetate extracts which suggest the potential of extracts as an anticancer agent [Figure 5].

### 4.5. Western Blotting and Activity Staining of SOD

**Figure 6:**
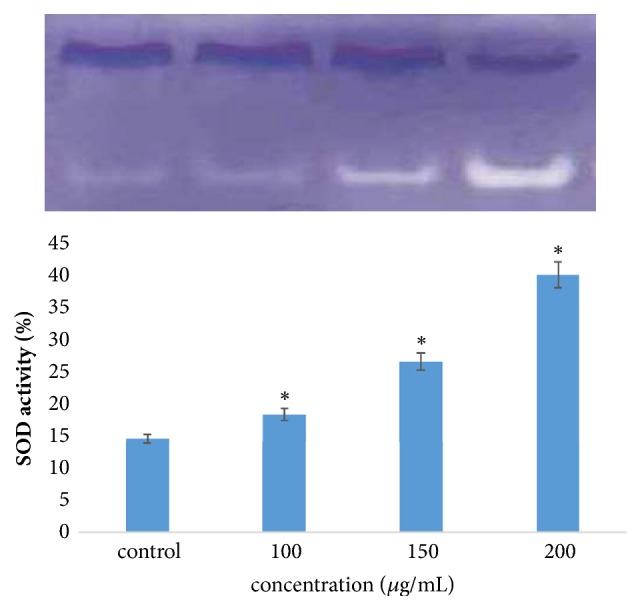
Effect of crude bacterial extracts on SOD (superoxide dismutase) in control and treated MCF-7 cells. Results are expressed in terms of values representing mean ± SD where n=3 at ^*∗*^P<0.05.

**Figure 7:**
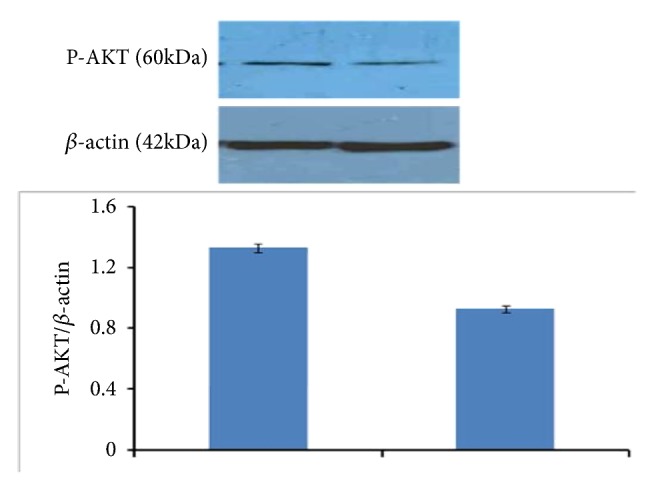
Effect of crude extract on activity of Akt in control and treated MCF-7 cells. Results are expressed in terms of values representing mean ± SD where n=3.

PI 3K, the direct upstream regulator of ser-threonine kinase Akt, is one of the frequently mentioned targets in human cancer. PI 3K phosphorylates phosphatidyl inositol and helps in attachment and activation of second messengers that control cellular activities and properties including proliferation, survival, and motility [36, 37]. Akt is one of the secondary downstream messenger protein which gets activation upon phosphorylation [38]. Phosphorylation of Akt leads to inhibition of proapoptotic signal as well as increase in antiapoptotic molecule [39]. Analysis through western blotting revealed that crude extract inhibits Akt activity which is noticed through reduced expression of phosphorylated Akt (thr 308) [Figure 7]. Reduced expression of phosphoAkt is expected to correlate with the increased expression of PTEN phosphatase which can maintain the level of Akt at this level [40]. Cell death or apoptosis is triggered by the presence of Reactive Oxygen Species (ROS). In the present study, it has been found that the level of most ubiquitous antioxidant enzyme SOD (superoxide dismutase) (here Cu-Zn SOD) which catalyzes the conversion/partitioning of superoxide radicals into hydrogen peroxide increased considerably in crude extracts treated cells in contrast to control [Figure 6]. Fragmentation analysis along with activity staining of SOD proves that crude ethyl acetate extract was efficient in killing cancer cells [41]. Earlier findings reported that decrease in Akt level and increase in SOD may be due to enhanced activity of PTEN tumor suppressor [38]. It can be assumed that the triggering factor is the degradation of NF*κ*-B which leads to the change in oxidative stress of MCF-7 cells upon treatment of extract [42–44]. Therefore, bacterial extract of* Bacillus anthracis* obtained from vermicomposting of sludge through the downregulation of Akt helps the cancer cells to take its last breath through nuclear degradation.

## 5. Conclusion

The present study has revealed a new dimension in the organic waste recycling process through vermitechnology with regard to its potential as an incubator of beneficial bacterial assemblages which can positively function and enrich the knowledge base in the field of health science. The ethyl acetate extract of bacteria performs a crucial role in controlling the growth of cancer cells. DNA fragmentation and nuclear fragmentation analysis along with MTT assay predict its role as an efficient facilitator of apoptosis. DPPH assay confirms its role as a good antioxidant in comparison with natural antioxidant ascorbic acid. Therefore, the present study can be considered as one of the pioneer studies which emerge a new way to prevent a disease like cancer using bacteria isolated from vermicomposting materials and thereby bring a new vista on multifarious uses of vermitechnology.

## Data Availability

The data used to support the findings of this study are available from the corresponding author upon request.
